# Linking Pathogenesis to Fall Risk in Multiple Sclerosis

**DOI:** 10.26502/aimr.0194

**Published:** 2025-01-30

**Authors:** Jaylan Patel, Marcel P. Fraix, Devendra K. Agrawal

**Affiliations:** 1 Department of Translational Research, College of Osteopathic Medicine of the Pacific, Western University of Health Sciences, Pomona, California 91766 USA.

**Keywords:** Cognition, Falls, Fall risk, Fatigue, Gait, Multiple sclerosis

## Abstract

Multiple Sclerosis is a chronic neurological disorder characterized by progressive disability, with falls being a significant consequence of its physical and cognitive impairments. This review explores the major contributors to fall risk in individuals with multiple sclerosis and explores the broader implications of these factors, such as the fear of falling. The primary factors associated with fall risk include gait abnormalities, cognitive dysfunction, and fatigue. These factors often interact, leading to mobility limitations and diminishing overall quality of life. Interventions to mitigate fall risk in multiple sclerosis have shown varying degrees of success. Exercise and rehabilitation strategies improve physical function and balance, while cognitive-behavioral therapy addresses fatigue and associated symptoms. Self-management programs empower patients to take an active role in symptom management, though their effectiveness varies. Disease-modifying therapies are the primary treatment for slowing disease progression, indirectly reducing fall risk. Emerging technologies show promise in enhancing mobility and safety, while machine learning algorithms offer the potential for predicting fall risk in multiple sclerosis populations. This review underscores the need for a comprehensive approach to fall prevention in multiple sclerosis. Healthcare providers can develop personalized strategies to improve mobility, reduce fall incidence, and enhance the quality of life for individuals with multiple sclerosis. Further research is essential to refine these interventions and optimize long-term outcomes.

## Introduction

Multiple Sclerosis (MS) is a chronic, progressive neurological disorder that affects the central nervous system, resulting in various symptoms that can impair mobility, cognition, and overall quality of life. The disease involves autoimmune-mediated demyelination and neurodegeneration, disrupting neural signaling and leading to neurological deficits. MS is more common in women and individuals of White race, with prevalence increasing globally due to several factors. Clinically, MS presents with a wide range of symptoms, including fatigue, cognitive dysfunction, and gait abnormalities. The disease presentation can vary, with there being different sub-types of MS depending on progression and remission. Risk factors are often genetic, environmental, and lifestyle based where each can play a large role in the development or progression of MS.

One of the major concerns for individuals living with MS is the increased risks of falls and their associated injuries, which are more common and worse due to the degenerative nature of the disease [3]. Mobility dysfunction in MS patients is frequently accompanied by gait instability, muscle weakness, and a loss of coordination, significantly hindering daily activities and reducing independence [4, 5]. This literature review will explore the complex relationship between MS-related mobility dysfunction and fall risk, examining the role of various factors such as cognitive impairment, fatigue, and the effectiveness of management strategies, including exercise, self-management programs, and pharmacological interventions.

### Pathophysiology, Epidemiology and Clinical Characteristics

#### Pathophysiology

**Figure 1:**
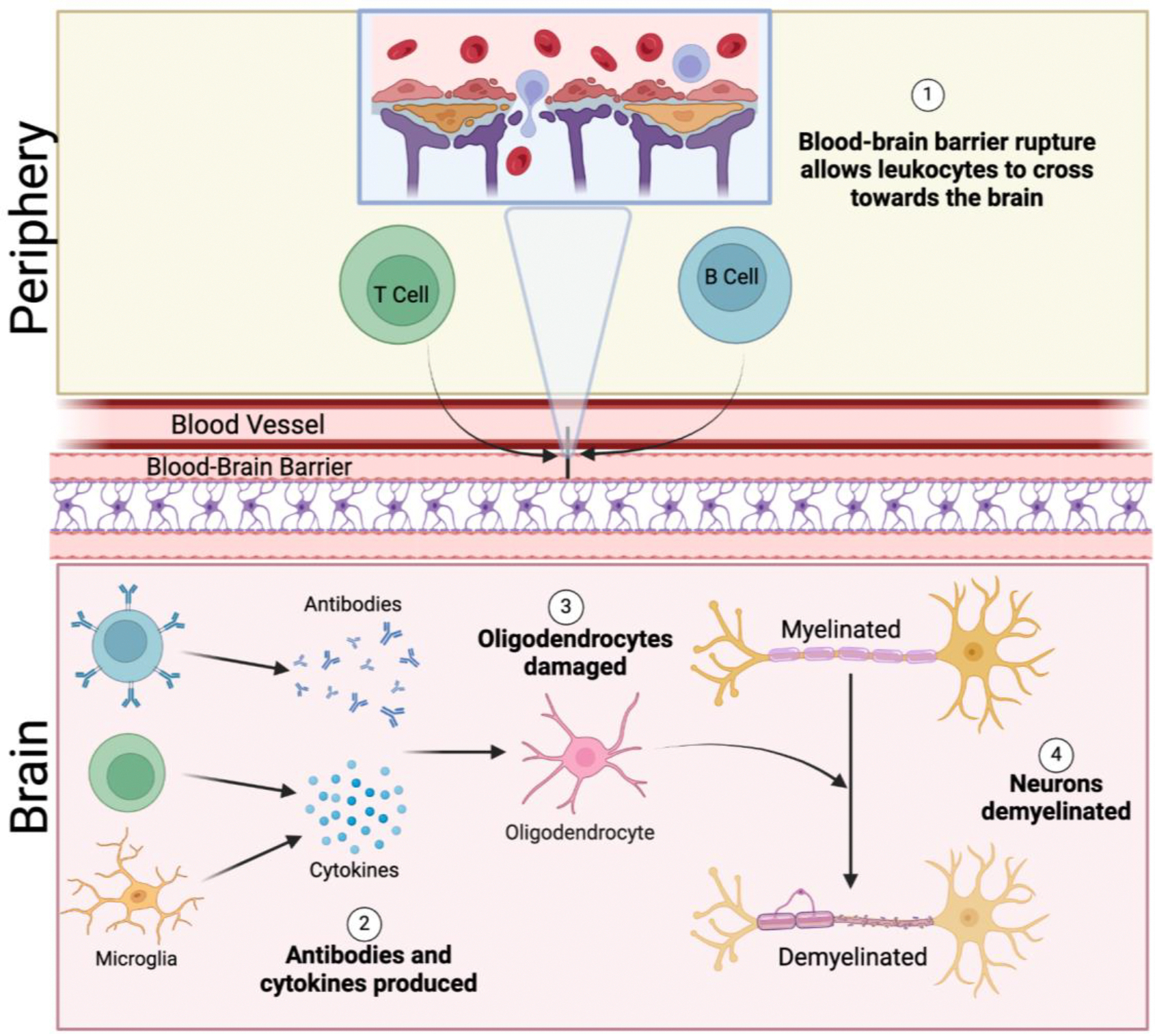
Schematic diagram showing the underlying cellular and molecular mechanisms in the pathogenesis of multiple sclerosis.

Multiple Sclerosis is an autoimmune disease where the immune system attacks myelin, the protective covering of nerve fibers, causing inflammation and nerve damage. This process begins by disrupting the blood-brain barrier, which normally prevents leukocytes from entering the brain and then triggers inflammation. Leukocytes, including T cells that produce cytokines and B cells that generate antibodies, are primarily responsible for demyelination, along with microglia, which contribute to inflammation [1]. These cytokines and antibodies attack oligodendrocytes, the cells responsible for producing myelin, ultimately damaging myelin and reducing nerve conduction [2]. This process is illustrated in Figure 1. The disease itself is primarily characterized by episodes of relapse and remission, with varying forms. The diverse nature of MS results in a broad spectrum of symptoms, including mobility impairments, cognitive dysfunction, and fatigue, all of which contribute significantly to the overall burden of the disease.

#### Epidemiology

It is important to identify the current prevalence of Multiple Sclerosis in today’s population and its change over time to determine the scope of healthcare needs and resource allocation. In a study estimating the prevalence of MS in the United States based on administrative health claims, data indicated that the prevalence of MS in adults has been steadily increasing, with 2010 marking an all-time high. Predictions for future trends of MS prevalence also suggest a continued increase [8]. This dataset was limited, as it did not include data from several U.S. communities, possibly indicating that the actual prevalence of MS could be higher than reported. This conclusion aligns with findings from studies in other countries that also show a rising prevalence of MS, potentially due to the aging population and increased incidence rates [17, 18]. Improved diagnostic techniques and heightened awareness among healthcare professionals may also contribute to the observed increase in reported cases. Additionally, changes in environmental and lifestyle factors, such as reduced physical activity and higher rates of obesity, might play a role in this trend. Recognizing these influences is essential for developing public health strategies and ensuring that medical infrastructure and resources are prepared to meet the growing demand for MS care. Understanding the factors contributing to increased prevalence will support better patient outcomes and more effective long-term management of the disease.

#### Clinical Presentation

Multiple Sclerosis is characterized by the destruction of myelin sheaths along neurons, which disrupts nerve signal transmission [4]. This progressive condition typically begins with neurological dysfunction and can evolve into chronic neurodegeneration, resulting in permanent neurological deficits. Clinically, MS manifests with a wide array of symptoms that vary based on the regions affected and the stage of the disease. Common symptoms include cognitive impairment, vision disturbances, sensory deficits, muscle weakness, and motor dysfunction [6, 7]. Certain populations are at a higher risk of developing MS, with studies indicating an increased prevalence among women and White individuals in the U.S. [8, 9]. Unique clinical features of MS include Lhermitte’s sign, an electric shock-like sensation triggered by neck flexion, and the Uhthoff phenomenon, where symptoms will worsen with elevated core body temperature [5, 6]. These diverse clinical presentations can lead to significant disability over time, affecting mobility, coordination, and the ability to perform daily activities. The variability and unpredictability of symptom progression make MS a challenging disease to manage, contributing to a heightened risk of falls and related complications.

#### Disease Progression

Multiple Sclerosis can be categorized into several types based on disease characteristics and progression. The most common form is Relapsing-Remitting Multiple Sclerosis (RRMS), which is characterized by episodes of neurological decline followed by complete or partial recovery, without continuous disease progression [10, 11]. This type is typically diagnosed in young adults and is influenced by factors such as relapse rates and environmental triggers. Over time, many individuals with RRMS transition to Secondary Progressive Multiple Sclerosis (SPMS) [11, 12]. SPMS follows the relapsing-remitting phase, especially in cases of inadequate treatment, and is marked by a progressive decline in neurological function, often leading to significant disability [10–12]. Primary Progressive Multiple Sclerosis (PPMS) is another major type, defined by a steady decline in neurological function from the onset, with no distinct relapses or remissions [10, 11]. Additionally, there is Clinically Isolated Syndrome (CIS), which refers to a single neurological episode suggestive of MS that does not yet meet diagnostic criteria. CIS often progresses to MS after subsequent episodes, with risk factors for conversion including neuronal demyelination and patient-specific factors such as body mass index (BMI) [13, 14]. However, further research is needed to clarify the mechanisms and predictors of CIS progression to MS. Each MS type can also be categorized as active or non-active, reflecting the presence or absence of ongoing relapses or disease progression. Active disease is associated with current relapses or new lesion formation, while non-active disease indicates a stable state. Despite this classification, progression independent of relapse activity has been identified as a major factor contributing to disability development across MS types [15, 16]. These classifications (Table 1) provide a framework for understanding the diversity of MS, encompassing its primary forms and clinical presentations within the disease spectrum.

#### Risk Factors

The exact cause of Multiple Sclerosis remains unknown, but it is widely believed to arise from a combination of genetic and environmental factors. Genetic predisposition plays a significant role in MS risk, as evidenced by studies examining familial and broader population-based heritability. A study involving 25,186 MS patients of Nordic ancestry assessed the heritability between PPMS and relapsing-onset MS. Results indicated a clear familial risk of developing MS. Although, no significant differences were found between the phenotypes, suggesting that similar mechanisms underlie different MS types. The highest risk was observed in full siblings, followed by parents and offspring [19, 20]. Further supporting this genetic association, a meta-analysis of genetic variants analyzed 32,367 MS cases alongside controls. This study identified a significant link between MS diagnosis and seven low-coding variants, emphasizing that low-frequency genetic variation contributes to MS heritability [21]. Understanding these genetic risk factors is crucial for determining the pathogenesis of MS and could guide the development of targeted therapies in the future.

Environmental and lifestyle factors also contribute to the development of MS. A case-control study found that adolescent smoking was linked to a significant increase in MS risk. The study also showed that a history of measles infection and a larger body size were associated with higher MS risk. At the same time, fish consumption and sunlight exposure have a protective effect [22]. This research underscores how complex the web of risk factors can be, touching on everything from infections to diet and lifestyle habits. However, the relatively small sample size means more extensive research is needed to confirm these findings and better understand their implications. In a genome-wide association study with 377,234 participants, physical activity was analyzed alongside the risk of Multiple Sclerosis. The results showed a statistically significant, negative correlation between physical activity and the risk of MS [23]. Intense physical activity during adolescence was associated with a decreased likelihood of developing MS, particularly when considering late-onset development. On the other hand, light physical activity did not demonstrate a significant association with MS risk in the same way as vigorous exercise [24]. This relationship is further supported by findings on obesity, which indicate a positive association between childhood obesity and an increased risk of developing MS later in life [25]. These studies highlight the importance of maintaining an active lifestyle and a healthy body weight from a young age to mitigate the risk of MS. Further studies would be useful to increase our understanding and strengthen these associations for possible preventative measures.

Smoking, in particular, stands out as a major risk factor not just for developing MS but also for increasing the chances of relapse. In a study of 355 MS patients undergoing treatment with natalizumab, those who smoked a pack a day had significantly higher relapse rates [26]. A similar study involving 834 patients treated with interferon-gamma found comparable results, showing an increased relapse rate among smokers, particularly those with relapsing-remitting MS [27]. These findings make it clear that lifestyle choices can have a real impact on disease progression. Encouraging smoking cessation could be a crucial part of managing MS and preventing relapses. It’s a reminder that while we often focus on treatments and medications, simple lifestyle changes can make a meaningful difference. Public health campaigns and support programs could play a vital role in helping MS patients make these changes and potentially improve their long-term health.

### Fall Risk and Contributing Factors in Multiple Sclerosis

**Figure 2:**
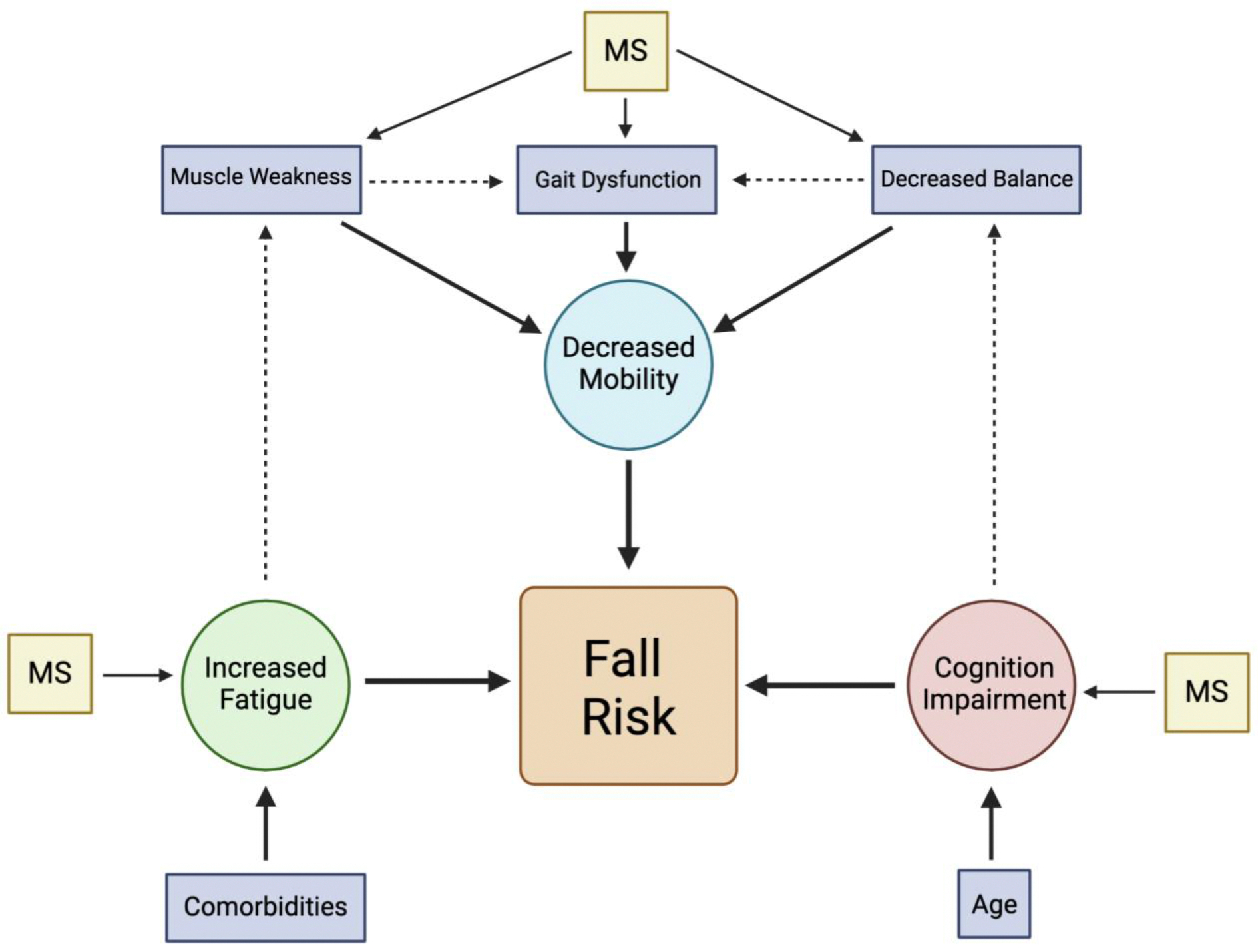
Flow chart illustrating the interconnected symptoms contributing to fall risk in patients with Multiple Sclerosis (MS).

Falls are a major concern for individuals with Multiple Sclerosis, posing an increased risk of injury and disability. Studies have consistently shown that those with MS experience a significantly higher incidence of falls compared to healthy controls, even after accounting for variables such as age and gender [28, 29]. This emphasizes the direct link between fall risk and the disease itself rather than external confounding factors. Figure 2 illustrates the major symptoms associated with increased fall risk in MS patients, which will be elaborated on in subsequent sections. Falls are particularly common among individuals with progressive MS, higher levels of disability, or impaired motor function [30]. While some falls may result in minor injuries, others can have severe consequences, especially for older adults, leading to serious injuries, disability, or loss of independence in activities of daily living [3]. Understanding the risk factors associated with falls in MS is essential for developing targeted preventive strategies to minimize injury and improve patient outcomes. Along with disability, a significant consequence of falls in MS is the risk of fractures. Low bone mineral density would increase the chances of fractures or breaks upon falling. Research indicates that individuals with MS frequently have reduced bone density and a higher prevalence of osteoporosis [31]. Alarmingly, population studies, such as one conducted in Canada, highlight low screening rates for bone density among individuals with MS, leaving many unaware of their elevated risk for fractures [32]. These findings demonstrate the importance of proactive bone health assessments and fall prevention measures in the MS population.

Predicting fall risk in individuals with Multiple Sclerosis is essential for preventing injuries. A study that utilized a machine learning algorithm to identify fall risk factors revealed several key contributors. The primary risk factors included the status of MS disease progression and the presence of disability. Smoking and exercise habits were identified as other influences of fall risk [33]. Monitoring the progression of MS in patients and assessing their level of disability are critical steps for physicians to evaluate the relative risk of falling. While interventions targeting disease progression and disability could have the greatest impact, these factors are largely beyond the patient’s immediate control. Therefore, promoting changes in smoking and exercise habits presents the most practical approach to reducing fall risk in individuals with MS.

Machine learning algorithms have been increasingly utilized to analyze gait data and assess fall risk in individuals with Multiple Sclerosis [34]. One study employed such algorithms to evaluate 11 different gait data sources, including gait analysis systems, walk tests, scales, and self-reported questionnaires. Among these, self-reported data emerged as the most reliable indicator of an individual’s health status. Self-reports of falls were shown to be strong predictors of future fall risk and the likelihood of injurious falls [34, 35]. However, this approach is limited by its retrospective nature, as it relies on a prior fall to generate predictions. It is difficult to integrate preventive interventions with this method. Furthermore, self-reports also possess other limitations. A study investigating their accuracy found discrepancies between actual and reported falls among individuals with MS, emphasizing the need for more reliable fall detection methods [36]. Larger studies are necessary to accurately evaluate the reliability of self-reports, in this context. Additionally, other machine learning algorithms have demonstrated high predictive accuracy by incorporating factors such as disability status, years since MS diagnosis, and demographic data [37]. An algorithm utilizing postural sway as a predictive measure has also shown promise in accurately assessing fall risk [38]. The variety of components contributing to fall risk has led to the development of numerous predictive models, making it challenging to determine which is most reliable. Enhancing these algorithms by integrating additional predictive factors could further improve their accuracy and support the development of more effective fall-prevention strategies for individuals with MS.

### Gait

Gait, or walking pattern, plays a critical role in evaluating an individual’s movement and fall risk. Abnormal gait is often associated with reduced motor function, making its assessment essential for understanding fall risk. Neurological disorders, such as Multiple Sclerosis, commonly lead to gait impairments [39], with severity influenced by various factors. A study on gait pathology in individuals with MS identified common deficits, such as reduced knee and ankle excursions [40]. Among these, impaired knee flexion has been highlighted as a significant dysfunction associated with MS, as noted in multiple studies [40, 41]. These gait abnormalities reflect the underlying pathology of MS, characterized by demyelination in the central nervous system, which results in muscle weakness and impaired motor control. Consequently, reduced motor function contributes to an increased risk of falls. Additionally, balance is often impaired in MS as well and is a critical factor in maintaining a stable gait. Its decline typically contributes to gait dysfunction and a heightened risk of falls [42].

An observational study investigated the relationship between spatio-temporal gait parameters and falls in individuals with Multiple Sclerosis [43]. Participants identified as “fallers” exhibited greater gait variability compared to the “non-faller” group, with step length variability showing the strongest correlation with fall risk, which is also supported by separate studies [43–45]. Further research found that fallers and near-fallers shared similar motor profiles, which differed from those of non-fallers [46]. These findings highlight the potential of gait analysis as a valuable tool for identifying individuals at higher risk of falls. Moreover, gait variability may represent a promising target for interventions aimed at reducing fall incidence in individuals with MS.

### Cognition

Cognitive impairment is a common and significant concern for individuals with Multiple Sclerosis, affecting various aspects of mental functioning and in turn affecting daily life. Studies have consistently shown that individuals with MS are at a significantly higher risk of cognitive impairment [47–49]. The most common deficits include reduced processing speed and difficulties with word finding [47, 48]. While these impairments are often less pronounced in younger individuals with MS, similar cognitive profiles observed in both younger and older populations suggest that these deficits are primarily a consequence of MS rather than age-related disorders [49]. Furthermore, the onset age of MS plays a role in the prevalence of cognitive impairment. Individuals with pediatric-onset MS are more likely to experience a rapid cognitive decline and a higher prevalence of cognitive impairment compared to those with adult-onset MS [50, 51].

Additionally, cognitive dysfunction is often associated with fall risk. It can negatively affect gait and balance, leading to an increased likelihood of falls. This relationship was demonstrated through self-reported falls and scales in individuals with MS, showing a clear correlation [52, 53]. Cognitive impairment in MS varies across disease stages [54] and is strongly linked to the mobility challenges associated with the condition. Therefore, monitoring cognitive function is crucial for accurately assessing fall risk. Proper evaluation of cognitive impairment enables physicians to identify patients at heightened risk, allowing them to provide targeted education and implement preventative strategies to reduce the likelihood of falls.

### Fatigue

Fatigue is a common and debilitating symptom associated with Multiple Sclerosis, significantly impacting daily activities and overall quality of life. Studies have consistently shown a strong association between fatigue and MS [55, 56]. Among the various forms of MS, progressive types are linked with higher levels of fatigue compared to non-progressive forms [57], and no significant difference in fatigue levels has been observed between the different subtypes of progressive MS [58]. Fatigue in MS is often made worse by comorbid conditions, which are prevalent in this population and contribute to worse clinical outcomes [59–61]. Depression and insomnia are among the most common comorbidities linked to fatigue [61, 62]. Research has shown that alleviating depression in individuals with MS leads to significant reductions in physical fatigue [63, 64]. Similarly, insomnia has been identified as a primary factor associated with fatigue in MS [65, 66]. Therefore, effective management of sleep disturbances and other comorbidities could substantially improve fatigue levels.

Fatigue is a critical factor in determining fall risk in individuals with MS. Utilizing self-reporting and fatigue scales, several studies have demonstrated a clear association between fatigue levels and fall risk among individuals with MS [67–70]. These findings emphasize the importance of identifying and addressing fatigue in this population to mitigate fall risk. Since fatigue contributes significantly to fall risk, managing comorbidities such as depression and insomnia not only improves fatigue but also reduces the likelihood of falls. Accurate assessment and targeted interventions for fatigue in individuals with MS can enhance safety, functional independence, and quality of life.

### Fear of Falling

Educating individuals with Multiple Sclerosis about their increased risk of falling and its potential consequences is crucial. However, this education can inadvertently contribute to a fear of falling, where individuals become overly cautious, limit movement, and adopt sedentary behaviors. A study employing a machine learning algorithm found that approximately 37% of individuals with MS experience a fear of falling, highlighting its relevance and prevalence within this population [71]. This fear often leads to reduced physical activity, which is associated with numerous adverse health outcomes, including an elevated risk of cardiovascular disease and diabetes [72, 73]. For patients with MS, maintaining adequate physical activity can be particularly challenging when their fear of falling is present.

Studies have shown individuals with a fear of falling often experience decreased physical activity, increased fatigue, and greater walking difficulties. Despite these challenges, there was no significant difference in fall incidence between individuals with a fear of falling and control groups [74, 75]. This highlights the negative impact of the fear of falling on overall well-being without a corresponding reduction in fall risk. Similarly, studies comparing fall incidence in active versus sedentary individuals with MS have found no significant difference between the two groups [76, 77]. This suggests that staying active and engaging in regular exercise does not increase the risk of falling for individuals with MS. On the contrary, maintaining an active lifestyle is essential for overall health. Current evidence does not support the notion that a sedentary lifestyle reduces fall risk, highlighting the need to encourage physical activity in individuals with MS. Therefore, patient education should emphasize both the risks of falling and the importance of staying active.

### Strategies to Reduce Fall Risk in Multiple Sclerosis

#### Exercise

As previously established, mobility impairment is a common issue for individuals with Multiple Sclerosis and can significantly impact daily activities. Therefore, managing this dysfunction alongside the progression of MS is crucial. Consistent exercise has been explored as a potential treatment for mobility impairments in MS patients. Several randomized controlled trials have evaluated various exercise methods, including high-velocity resistance training, intense aerobics, progressive resistance training, and cycling. Some of these studies reported statistically significant improvements in balance, walking ability, muscle strength, and fatigue [78–80]. These findings suggest that exercise may be an effective treatment for mobility dysfunction in MS. Studies also show evidence of increased cognitive processing speed, potentially due to improved cardiorespiratory fitness [81]. This cognitive improvement may contribute to a reduced risk of falls. However, other studies have found no significant evidence of walking improvement among individuals actively exercising compared to control groups. Instead, the most consistent benefits observed were enhanced muscle performance and reduced fatigue in the exercise group [82–86]. Despite mixed results regarding walking improvements, most studies reported a correlated increase in the quality of life for participants [78–80, 82–84]. Additional research is needed to further explore the exact effects of exercise on mobility dysfunction in individuals with MS. Notably, innovative interventions like exoskeleton-assisted walking have shown promising results. One study demonstrated a significant improvement in functional mobility with exoskeleton-assisted walking compared to conventional gait training [87]. This method holds potential for individuals with mobility and cognitive impairments contributing to gait dysfunction. Improving gait through such interventions could reduce fall incidence in individuals with MS, offering a meaningful approach to enhancing both mobility and overall quality of life.

#### Self-Management Programs

Self-management programs are another approach being explored as a treatment for mobility dysfunction in individuals with Multiple Sclerosis. These programs are designed to educate participants and empower them to achieve specific health outcomes. Some self-management programs focus on reducing fall incidence in individuals with MS by combining education and exercise over a set period. One such program, “Free from Falls”, demonstrated a relative decrease in fall incidence among participants compared to those who received only a brochure. However, the reduction in falls was only slightly better than the control group [88]. A similar study testing the same program found no significant difference in fall incidence between participants and those receiving neurologist-initiated interventions and education [89]. These findings suggest that the efficacy of the “Free from Falls” program is comparable to standard physician-led education, highlighting the need for further evaluation. Another program, Balance Right in MS, focuses on improving balance as a means of reducing fall incidence and is currently undergoing testing to determine its effectiveness [90]. Additional research is required to refine existing programs or develop new ones to achieve the desired outcome of reducing falls among individuals with MS.

Various programs have been developed to assist in managing the diverse symptoms associated with Multiple Sclerosis, including fatigue. Self-management programs that combine education and symptom management strategies have demonstrated effectiveness in reducing fatigue in individuals with MS [91–93]. However, like the fall prevention program, studies indicate that these programs may not consistently outperform general patient education in achieving desired outcomes [94]. While fatigue reductions were reported, the differences between the program and general advice were not statistically significant. Despite this, self-management programs may still hold value, particularly in cases where patient education is insufficient or inaccessible. They offer structured guidance that could benefit individuals who require additional support in managing their symptoms.

#### Cognitive Behavioral Therapy

Cognitive Behavioral Therapy (CBT) is a type of psychotherapy widely utilized for managing symptoms associated with Multiple Sclerosis. Fatigue is a significant contributor to fall risk in MS, and several studies have demonstrated that CBT is effective in reducing MS-related fatigue [95–98]. Notably, evidence suggests a stronger association between fatigue reduction and CBT than with exercise interventions in MS patients [98]. By alleviating fatigue, CBT may contribute to a reduction in fall risk among individuals with MS. CBT also addresses other common MS symptoms, such as pain and insomnia, which can indirectly influence fall risk. Pain, often debilitating in MS patients, may be mitigated with CBT, though further research is required to confirm its efficacy in this context [99]. Insomnia, another prevalent symptom of MS, has been shown to improve significantly with CBT in the general population [100–102], with similar benefits observed in MS patients [103]. Improved sleep quality from CBT could lead to reduced fatigue, thereby decreasing fall risk. Overall, CBT presents a versatile treatment strategy for managing multiple MS symptoms, highlighting its potential role in comprehensive fall risk management. Further research is needed to explore its broader applications in MS care.

#### Medication

Medication is often the primary form of treatment for Multiple Sclerosis, with disease-modifying therapies (DMT) playing an important role in reducing disease activity and slowing progression. Fumarate, an oral DMT, is commonly prescribed for active RRMS and has demonstrated efficacy in reducing relapse rates. However, gastrointestinal side effects are frequently reported [104, 105]. Interferon beta, an injectable DMT, is also effective in managing relapsing forms of MS by reducing relapse frequency and severity. It also can cause side effects, including flu-like symptoms and injection site reactions [106]. Early initiation of DMTs before an MS diagnosis has been shown to lower the risk of a first clinical episode, emphasizing the importance of timely diagnosis and intervention [107]. Additional research is required on the treatment’s effect in the context of early application. Despite their effectiveness, DMTs are not targeted for the later, progressive forms of MS, such as PPMS and SPMS, where limited and conflicting research highlights the need for further investigation.

A significant challenge to DMT efficacy is patient compliance, with studies showing that approximately 31% of patients fail to adhere to prescribed treatments due to personal beliefs or outlooks [108]. Addressing noncompliance through targeted interventions and identifying contributing factors is essential for maximizing the benefits of DMTs. By slowing disease progression and preserving motor function, these therapies can inherently reduce fall risk in individuals with MS. A study assessing fall risk in patients with MS found that the group receiving DMT treatment had a 48% decreased incidence of falling compared to those not receiving treatment [109]. This suggests that DMTs may not only indirectly impact fall risk by slowing disease progression but can also directly influence fall incidence. The benefits of these therapies are maximized when patients begin treatment early and follow prescribed regimens consistently. Further research is essential to evaluate the long-term impact of DMTs on fall risk, particularly in patients with advanced MS, and to identify strategies that can enhance patient compliance and optimize therapeutic outcomes.

## Figures and Tables

**Table 1:** Subtypes of Multiple Sclerosis and their associated features.

Subtype	Characteristics	Disease Progression	Additional Notes
**Clinically Isolated Syndrome**	A single episode of MS-like symptoms that does not meet the criteria for an MS diagnosis.	May progress to MS after further episodes.	BMI and demyelination are risk factors for progression to MS
**Primary Progressive**	Consistent decline in neurological function from the onset, with no relapses or improvement.	Continuous progression from onset.	
**Relapsing-Remitting**	Episodes of neurological degradation followed by complete or partial recovery	No continuous progression during remission.	Most common form, frequently diagnosed in women and individuals of White race.
**Secondary Progressive**	Follows RRMS, marked by a progressive decline in neurological function, often resulting in disability.	Gradual worsening over time.	Transition often occurs when treatment during RRMS is insufficient.

BMI, body mass index; MS, multiple sclerosis; RRMS, relapsing-remitting multiple sclerosis

**Table 2:** Treatments for Multiple Sclerosis with their pros, cons, and impact on fall risk

Treatment	Pros	Cons	Fall Risk Impact
**Exercise**	- Improves strength, balance, fatigue - Enhances quality of life	- Mixed evidence for walking gains - Risk of injury without guidance	- Reduces fall risk via mobility gains - Essential for independence
**Cognitive Behavioral Therapy**	- Reduces fatigue & insomnia - Addresses depression & pain	- Requires trained therapists - Limited research for some symptoms	- Reduces fatigue, improving safety - Better sleep, mood
**Self-Management Programs**	- Provides education & empowerment - Addresses fatigue & comorbidities	- Mixed results vs. general advice - Success depends on engagement	- Raises awareness & reduces risk - Useful with limited care access
**Disease Modifying Therapies**	- Manages disease directly - Slows disease progression	- Side effects - Patient compliance is important	- Variable impact on fall risk - Symptom relief may lower risk

## Key Points

- Multiple sclerosis can result in a range of physical and cognitive impairments, contributing to mobility dysfunction, cognitive deficits, fatigue, and an increased risk of falls.
- Gait dysfunction in multiple sclerosis is a significant factor contributing to fall risk, with impairments often resulting from a combination of factors, such as muscle weakness, cognitive dysfunction, and fatigue.
- Cognitive impairments in MS are associated with both disease progression and fall risk, affecting gait and balance, and increasing fall likelihood in patients.
- Fatigue is a prevalent symptom in MS, increasing mobility dysfunction and contributing to fall risk, especially when combined with comorbidities, such as insomnia, where effective treatment of these comorbidities can help alleviate fatigue and reduce fall risk.
- Exercise interventions have shown benefits in improving muscle performance, balance, and reducing fatigue, though the direct impact on mobility in MS patients remains mixed.
- Self-management programs, particularly those focusing on fall prevention and combining education with exercise, have demonstrated some success, but their efficacy is often like physician-led approaches.
- Cognitive behavioral therapy has proven effective in reducing MS-related fatigue, pain, and insomnia, indirectly reducing fall risk through their alleviation.
- Disease-modifying therapies slow disease progression and reduce fall risk by preserving motor function, but patient noncompliance remains a significant challenge to their efficacy.
